# Herbage allowances during mid to late gestation affect growth performance and physiological responses of beef cow–calf pairs, and offspring skeletal muscle development

**DOI:** 10.1007/s11250-026-05035-4

**Published:** 2026-05-08

**Authors:** William Luiz de Souza, Luciana Melo Sousa, Iorrano Andrade Cidrini, Germán Darío Ramírez-Zamudio, Karla Alves Oliveira, Ivanna Moraes de Oliveira, Laura Franco Prados, Gustavo Rezende Siqueira, Flávio Dutra de Resende

**Affiliations:** 1 Department of Animal Science, São Paulo State University, Jaboticabal, SP 14884-900 Brazil; 2 Department of Animal Science and Food Engineering, São Paulo University, Pirassununga, SP 13635-900 Brazil; 3 Department of Animal Sciences, North Dakota State University, Fargo, ND 58108 USA; 4 Agência Paulista de Tecnologia dos Agronegócios, Colina, SP 14770-000 Brazil; 5 Institute for Future Farming Systems, Central Queensland University, Rockhampton, QLD 4701 Australia

**Keywords:** Fetal programming, Cow-calf industry, Pasture-based system, Offspring skeletal muscle development, Restricted nutrition

## Abstract

Our objectives were to determine the effects of two herbage allowance levels during mid to late gestation on growth performance, physiological responses of cow–calf pairs, and offspring skeletal muscle development through weaning. Fifty-six pregnant Nellore cows [444 ± 42 kg initial shrunk body weight (SBW) and 3.66 ± 0.28 body condition score (BCS)], each carrying a male fetus, grazed *Urochloa brizantha* cv. Marandu pastures under either low HA (LHA; 2.80 kg dry matter (DM)/kg BW) or high HA (HHA; 7.60 kg DM/kg BW) during the final 151 days of gestation (d 140 ± 15 of gestation). As intended, differences in HA (*P* < 0.01) were established by manipulating stocking rate (*P* < 0.01; 1.70 vs. 3.40 AU/ha for HHA and LHA, respectively). LHA cows had lower body weight (BW; *P* < 0.001), average daily gain (ADG; *P* < 0.001), BCS (*P* < 0.001), *Longissimus* muscle area (LMA; *P* = 0.002), and subcutaneous fat thickness (SFT; *P* = 0.02) than HHA cows during gestation. During lactation, LHA and HHA cows showed similar BW, LMA, and SFT (*P* ≥ 0.12); however, LHA cows had greater ADG (*P* < 0.001) and lower BCS (*P* < 0.001) than HHA cows. In late gestation, LHA cows exhibited higher plasma urea (*P* < 0.01) than HHA cows, but no differences were detected in milk yield or composition during lactation (*P* ≥ 0.20). HHA calves had greater BW at birth (*P* < 0.01), at 120 days of age (*P* < 0.01), and at weaning (*P* < 0.01) than LHA calves. HHA calves also had more muscle fibers at 30 (*P* < 0.01) and 240 (*P* < 0.01) days of age than LHA calves, whereas muscle-fiber cross-sectional area did not differ at either 30 or 240 days days of age (*P* ≥ 0.21). In conclusion, adequate herbage allowance during mid- to late gestation improves maternal performance and metabolic status, supporting fetal skeletal muscle development and enhancing offspring growth through weaning.

## Introduction

Pasture systems serve as the foundation for diverse calf-raising operations worldwide (Greenwood 2021). However, these systems face the seasonality of herbage allowance throughout the year, experiencing swings in both herbage quantity and quality (Roth et al. 2017; Sousa et al. 2024). For instance, summer conditions favor herbage production, providing an optimal environment for cattle rearing in pastures and enabling grazing intensity strategies. Nonetheless, adverse conditions in winter necessitate specific strategies to surmount challenges in herbage growth (Wingler and Hennessy 2016; Brown et al. 2019).

It is commonplace to encounter animals with nutritional deficiencies during periods of herbage scarcity (Barbero et al. 2020), among the categories impacted by the seasonality of herbage production, pregnant beef cows stand out. It is crucial to emphasize that adequate nutrition for gestating cows is essential not only for their own maintenance and fetal development but also to ensure the productive performance of their progeny throughout its life cycle (Brandão et al. 2020; Sousa et al. 2024; Perry and Welsh Jr 2024). Beyond immediate effects, maternal nutrition plays a critical role in shaping the long-term developmental trajectory of the offspring, a process referred to as fetal programming (Du et al. 2010; Costa et al. 2021a). During mid- to late gestation in beef cows, specific nutritional conditions may induce maternal tissue mobilization, particularly of skeletal muscle, resulting in the release of glucogenic amino acids that can be utilized as substrates for hepatic gluconeogenesis (Bell and Ehrhardt 2000), thereby contributing to maternal glucose supply and supporting adequate fetal development (Moreira et al. 2021; Redifer et al. 2023).

To address these periods of nutrition restriction, Costa et al. (2021b) underscore that strategic supplementation at different gestational stages can enhance muscular development and offspring performance. In this regard, several studies highlight that supplementing pregnant cows to prevent periods of nutritional restriction, particularly during mid-to late gestation, promotes greater muscle fiber formation in the fetus and improves postnatal growth and performance (Du et al. 2010; Márquez et al. 2017; Meresca et al. 2019; Valiente et al. 2019; Rodrigues et al. 2020; Nascimento et al. 2022; Nascimento et al. 2024; Cidrini et al. 2025). These findings underscore the strategic importance of supplementation in optimizing offspring growth and development.

However, with supplementation costs can be relatively high in beef calf production systems (Souza et al. 2023). Pasture management, with a focus on herbage allowance, can improve the productivity of calf-rearing systems (Do Carmo et al. 2018; Sousa et al. 2024). However, knowledge about herbage allowance for pregnant cows in tropical regions is limited, since land with more marginal productivity for beef production is allocated to breeding herds worldwide (Greenwood 2021). This can restrict the availability and quality of pasture needed to ensure adequate nutrient intake. Likewise, the effects on cow–calf performance and on the muscular attributes of the progeny, especially in *Bos indicus* herds under low or no supplementation, remain poorly understood.

Given that, we hypothesize that a high herbage allowance during the mid- to late gestational periods improves the metabolic state and body condition of cows during gestation, resulting in calves with greater growth due to alterations in muscle fibers during fetal life. Our objectives were to determine the effects of two herbage allowance levels during mid- to late gestation on growth performance, physiological responses of cow–calf pairs, and offspring skeletal muscle development until weaning.

## Materials and methods

**Fig. 1:**
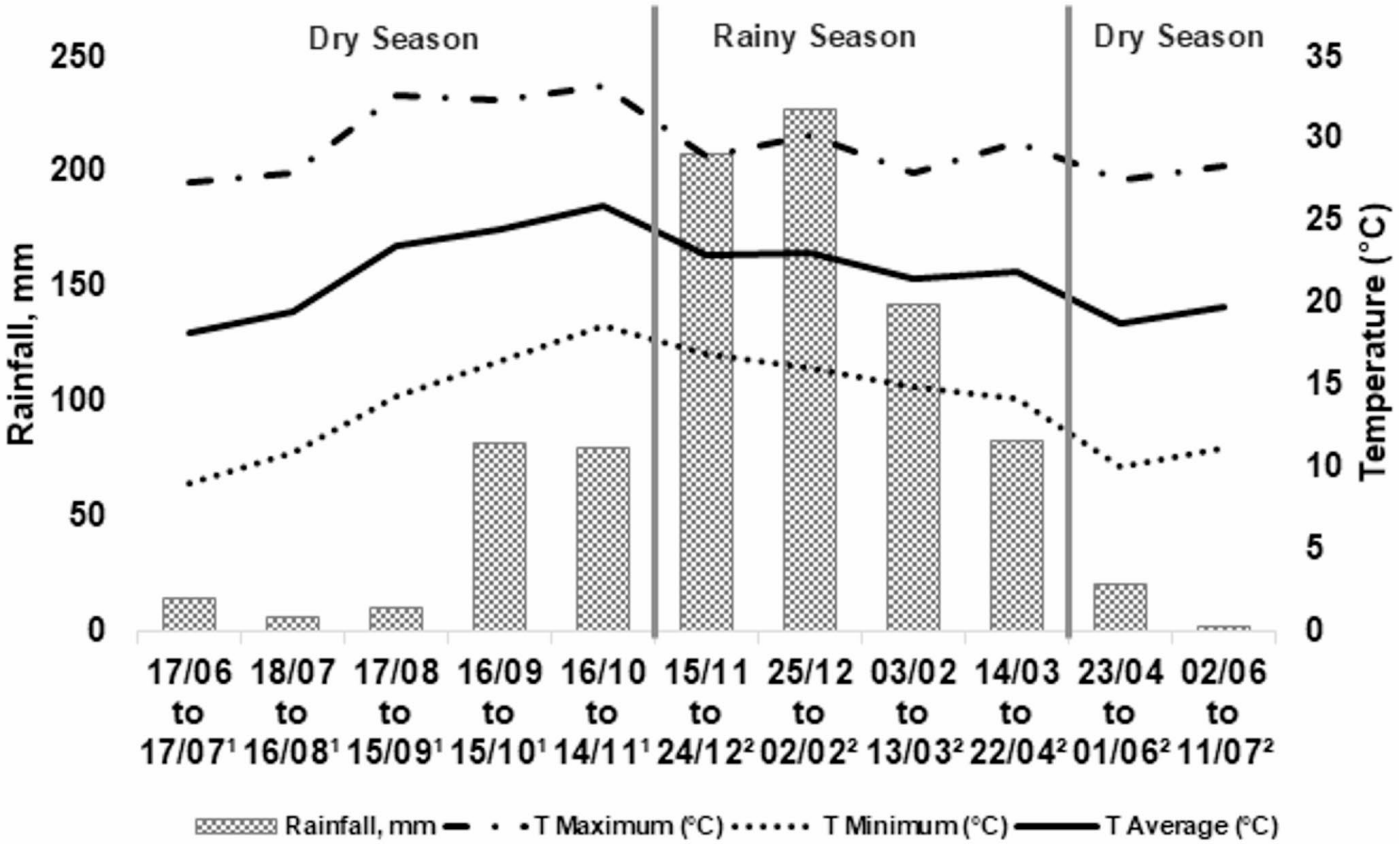
Climatic data from the beginning of treatment application to the completion of the experiment, totaling 390 days. Superscript ¹ indicates the gestation period evaluated, and superscript ² indicates the lactation period

The study was carried out at the Agência Paulista de Tecnologia dos Agronegócios (APTA), Colina, São Paulo, Brazil. All animal procedures were approved by the APTA Animal Ethics Committee (protocol 0004/2020) and conducted in accordance with institutional and national guidelines for the care and use of animals. The climate data from the experiment are presented in Fig. 1.

### Animals, experimental design, and treatments

Four hundred and fifty multiparous Nellore cows (452 ± 38 kg of bodyweight (BW) and 5 ± 2 calvings) were subjected to a fixed-time artificial insemination protocol using three different Nellore bulls. Pregnancy was diagnosed 30 days after insemination, and fetal sex was determined on gestational day 60 by ultrasonography (7.5-MHz transducer; Aloka SSD-500; Aloka, Tokyo, Japan).

**Fig. 2:**
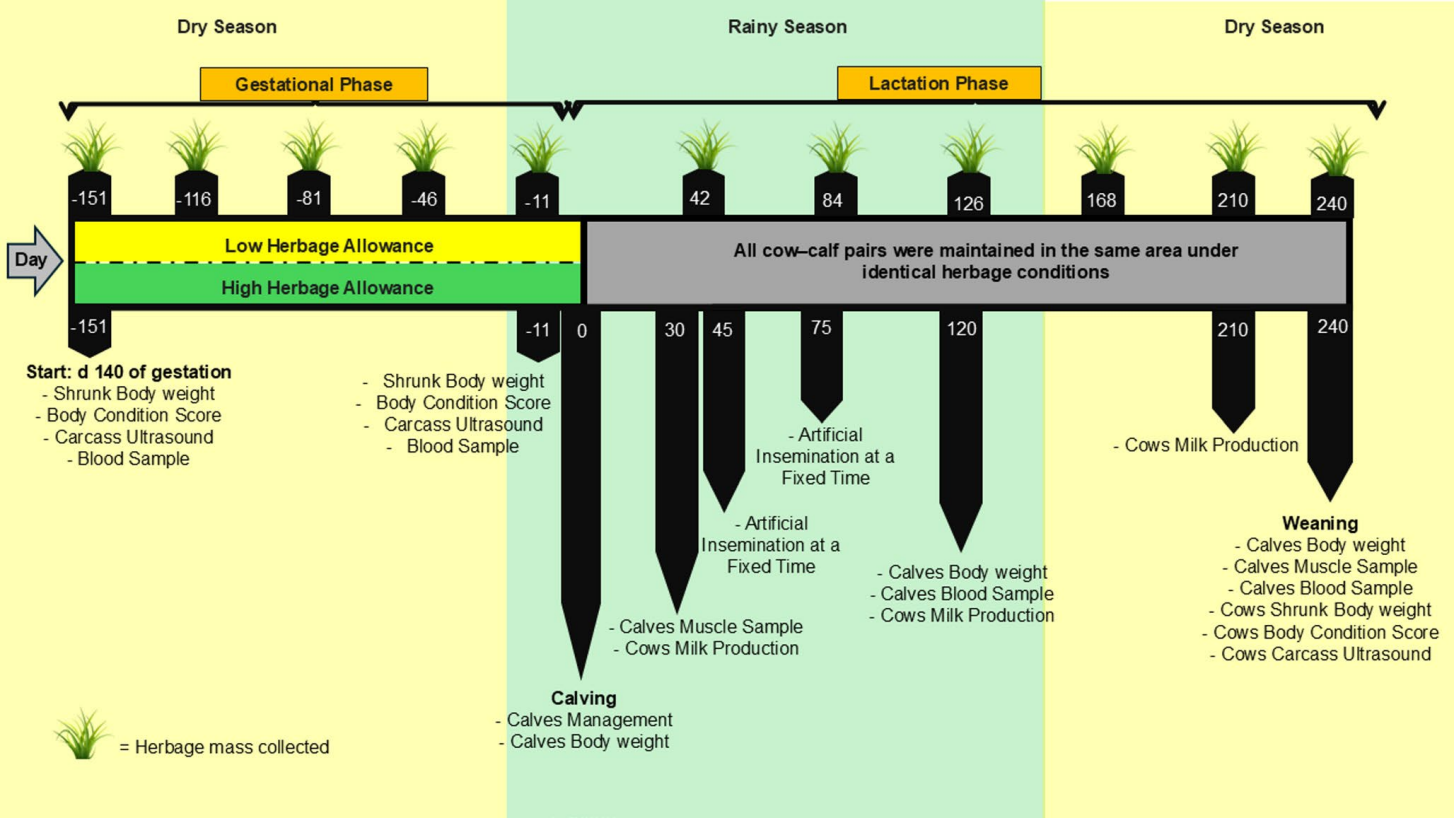
Experimental timeline

Fifty-six cows confirmed to be carrying male fetuses were enrolled, at baseline, cows averaged 444 ± 42 kg shrunk body weight (SBW) and 3.66 ± 0.28 body condition score (BCS; 1–5 scale; Houghton et al. 1990). The experiment followed a randomized complete block design, with SBW used as the blocking factor, with six blocks and 12 experimental units. Each unit comprised a 7.5-ha paddock, totaling 90 ha of *Urochloa brizantha* cv. Marandu pasture. All paddocks were equipped with water troughs and feeders for supplementation. Treatments were based on maximum and minimum herbage allowances, where low herbage allowance (LHA) was defined as a maximum of 3.50 kg dry matter (DM)/kg BW, while high herbage allowance (HHA) was set as a minimum of 6.50 kg DM/kg BW. Treatments were applied from d − 151 of experiment (d 140 ± 15 of gestation) to d 0 (calving season), with the final mean values for pasture allowance being as follows: (1) LHA: 2.80 kg DM/kg BW or (2) HHA: 7.60 kg DM/kg BW (Fig. 2).

During gestation, all cows received a protein supplement at a rate of 1 g/kg BW. The supplement had the following composition on a DM basis: 920 g/kg DM; 500 g crude protein (CP)/kg DM; 330 g total digestible nutrients (TDN)/kg DM; and 660 g mineral mixture/kg DM. The mineral mixture contained: 50 g calcium (max); 35 g calcium (min); 20 g phosphorus (min); 80 g sodium (min); 15 g sulfur (min); 5000 mg magnesium (min); 45 mg cobalt (min); 400 mg copper (min); 25 mg iodine (min); 260 mg manganese (min); 9 mg selenium (min); 1700 mg zinc (min); and 200 mg fluorine (max). A fixed stocking rate was applied during continuous grazing in the gestation period to establish contrasting herbage allowances, resulting in two stocking densities: 3.40 AU/ha in the LHA and 1.70 AU/ha in the HHA. Continuous grazing was managed with a variable stocking rate using a put-and-take system for cow–calf pairs during lactation, with non-lactating cows (without calves) added or removed as needed to maintain a target sward height between 15 and 30 cm (Mott and Lucas 1952). All animals were moved to a single 15-ha pasture with an average herbage mass of 7146 ± 1980 kg DM/ha and CP concentration of 67.1 ± 9.0 g/kg DM. A mineral salt mixture was provided *ad libitum* to cow–calf pairs until weaning and contained (on a DM basis): 990 g/kg DM; 185 g calcium (max); 160 g calcium (min); 80 g phosphorus (min); 107 g sodium (min); 12 g sulfur (min); 5000 mg magnesium (min); 107 mg cobalt (min); 1300 mg copper (min); 70 mg iodine (min); 1000 mg manganese (min); 18 mg selenium (min); 4000 mg zinc (min); and 800 mg fluorine (max).

### Pasture measurements

Herbage mass was assessed during gestation every 35 d and during lactation every 42 d using the method described by Sollenberger and Cherney (1995) that a total of 100 canopy height readings were taken with the aid of a rising plate meter. Herbage samples were collected at ground level from three points, minimum, average, and maximum height (determined based on ± 2 standard deviations from the mean), in a specified area of 0.25 m² in each paddock. The samples were oven-dried at 55 °C with forced air circulation for 72 h to obtain partial DM. Finally, linear regression equations were developed using herbage mass from the different collection points to establish the relationship between canopy height and herbage mass.

To characterize herbage composition, herbage samples were collected by hand-plucking (De Vries 1995) before biomass harvest. Samples were dried in a forced-air oven at 55 °C for 72 h and then ground in a knife mill (MA680; Marconi, Piracicaba, Brazil) to pass 2- and 1-mm screens. Proximate analyses followed AOAC (2004): DM (934.01), ash (942.05), CP (978.04), and ether extract (920.39). Organic matter (OM) was calculated as DM minus ash. Neutral detergent fiber (NDF; INCT-CA method F-013/1) and acid detergent fiber (ADF; INCT-CA method F-015/1) were determined using an adapted procedure based on Detmann et al. (2021), with analyses performed in a fiber analyzer (TE-149; Tecnal, Piracicaba, São Paulo, Brazil). In vitro digestibility was measured as described by Holden (1999). Indigestible NDF (iNDF) was quantified after 288 h ruminal incubation of 2-mm ground samples in rumen-cannulated cattle (Valente et al. 2011).

### Growth performance and carcass ultrasound measurements

Cows were weighed, had BCS assessed, and underwent carcass ultrasound on d -151, d -11, and d 240. Performance was evaluated by calculating average daily gain (ADG), determined by the difference between the final shrunk BW (after 16 h of solids and liquids restriction) and the initial SBW, and the value was obtained by averaging over the number of days in the experimental period. Additionally, BCS on a scale of 1 to 5, subdivided into 0.25-point intervals, was determined by two trained evaluators following the methodology of Houghton et al. (1990). Two artificial insemination at a fixed time protocols were conducted on d 45 and d 75 to determine the final pregnancy rate of cows subjected to different treatments.

Variables measured via ultrasonography included *longissimus* muscle area (LMA, cm²) and subcutaneous fat thickness (SFT, mm) in the *longissimus* muscle between the 12th − 13th ribs and the ultrasonography was performed using an ultrasonic device (Aloka^®^ SSD-500 Vet, Tokyo, Japan) fitted with a 3.5-MHz linear probe and an acoustic guide. Calves were weighed at three distinct times during the experiment: On calving, d 120, and weaning, the final weaning BW adjusted for 205 days of age. Newborn calves were weighed, identified via tattoo, and dewormed with 1 ml of 1% Doramectin per 50 kg BW (Dectomax^®^ injectable; Zoetis, Guarulhos, Brazil). The ADG for the calves was calculated according to each period.

### Milk yield and composition

Eighteen cows per treatment were randomly selected to estimate average milk yield. Milking occurred on d 30, d 120, and d 210. Calves were separated from the cows at 17:00 h, and milking was performed at 05:00 h on the following morning. Milking start time was recorded, and 24 h yield was estimated using equation. Half of the cows were milked daily within a 3 h window using a mechanical milking machine operated by trained personnel. Milk ejection was induced through the administration of 2 mL of oxytocin (10 IU/mL; Ocitovet^®^, Vet & Cia Saúde Animal, São Paulo, Brazil). After milking, milk was homogenized, weighed (± 10 g), and recorded for 24 h yield was estimated using the Eq. 1, proposed by Restle et al. (2003):

1 $$\mathrm{M}\mathrm{Y}\:(\mathrm{k}\mathrm{g}/\mathrm{d})=\:\frac{\mathrm{M}\mathrm{m}\mathrm{Y}\:\left(\mathrm{k}\mathrm{g}\right)\:\times\:24\:\mathrm{H}\mathrm{o}\mathrm{u}\mathrm{r}\mathrm{s}}{\mathrm{M}\mathrm{m}\mathrm{T}}$$

Where MY - daily milk yield (kg/day), MmY - milk yield at morning milking, and MmT - the time of the recorded morning milking of the cows, subtracting the time at which the calves were separated from the cows on the previous day, to obtain the time interval between these two events.

The recorded milk yield was expressed as 4% fat-corrected milk (4% FCM) using the Eq. 2 proposed by the NRC (2001):

2 $$\mathrm{4}\:\mathrm{\%}\:\mathrm{F}\mathrm{C}\mathrm{M}\:(\mathrm{k}\mathrm{g})\:=\:[0.4\:\times\:\:\mathrm{m}\mathrm{i}\mathrm{l}\text{k }\:\mathrm{y}\mathrm{i}\mathrm{e}\mathrm{l}\mathrm{d}\:\left(\mathrm{k}\mathrm{g}\right)]\:+\:[15\:\times\:\:\mathrm{f}\mathrm{a}\mathrm{t}\:\text{ y}\mathrm{i}\mathrm{e}\mathrm{l}\mathrm{d}\:\left(\mathrm{k}\mathrm{g}\right)]\:$$

A milk sample from each of the eighteen cows was collected in specific containers containing a bromopol preservative (2-bromo-2-nitropropan-1,3-diol; D&F Control Systems^®^, Inc., Dublin, CA). The samples were refrigerated and then sent to the Milk Analysis Laboratory (Clínica do Leite, Piracicaba, SP, Brazil). The following characteristics were assessed: milk fat (%), milk protein (%), milk lactose (%), milk total solids (%), milk casein (%), and milk urea (mg/dL). These analyses were performed using infrared spectroscopy with the MilkoScan FT1 analyzer (Foss, Hillerod, Denmark). Somatic cell count was determined using the flow cytometric method with the Bentley Combi System 2300^®^ (Bentley Instruments Incorporated, Chaska, USA).

### Blood collection and analysis

Eighteen cows were randomly selected from each treatment for blood collection via jugular vein puncture, conducted on d -151 and d -11. Additionally, eighteen calves per treatment were blood sampled on d 120 and at weaning. These calves were the offspring of the cows that had also provided blood samples, and the blood was collected using Vacutainer^®^ tubes. Blood samples were centrifuged at 3,000 × g for 10 min at 4 °C; plasma was separated and stored at − 20 °C until analysis. Albumin (K040; Bioclin^®^ Quibasa, Belo Horizonte, Brazil) was quantified by a bromocresol green colorimetric method; aspartate aminotransferase (K048; Bioclin^®^ Quibasa, Belo Horizonte, Brazil) by a kinetic UV method; gamma-glutamyl transferase (K080; Bioclin^®^ Quibasa, Belo Horizonte, Brazil) by a modified Szasz method standardized by IFCC (kinetic); glucose (K082; Bioclin^®^ Quibasa, Belo Horizonte, Brazil) by an enzymatic colorimetric method; total cholesterol (K083; Bioclin^®^ Quibasa, Belo Horizonte, Brazil) by an enzymatic colorimetric method; triglycerides (K117; Bioclin^®^ Quibasa, Belo Horizonte, Brazil) by an enzymatic colorimetric method; creatinine (K016; Bioclin^®^ Quibasa, Belo Horizonte, Brazil) by a modified Jaffé colorimetric method; total protein (K031; Bioclin^®^ Quibasa, Belo Horizonte, Brazil) by the biuret colorimetric method; and urea (K056; Bioclin^®^ Quibasa, Belo Horizonte, Brazil) by a urease/GLDH kinetic UV method. Absorbance readings were obtained within the range specified for each kit using an automatic biochemistry analyzer (SBA-200; CELM^®^, Barueri, São Paulo, Brazil), which performs endpoint and kinetic photometric reactions. Plasma IGF-1 and insulin were measured by chemiluminescent enzyme immunoassay using a commercial platform (Immulite^®^ 1000; Siemens Healthcare Diagnostics, Munich, Germany) at the Laboratory of Animal Nutrition and Growth (LNCA, ESALQ/USP, Piracicaba, SP, Brazil).

### Skeletal muscle sampling and histology

Samples were collected from the *longissimus* muscle at the level of the 12th–13th ribs via biopsy in calves at d 30 and d 240. For the biopsy, the animal’s dorsal area was previously sanitized with 70% ethanol, followed by the application of local anesthesia based on 2% lidocaine hydrochloride without epinephrine (Lideovet, Bravet LTDA, Rio de Janeiro, RJ, Brazil). After ensuring insensitivity in the biopsy area, a parallel incision to the muscle was made, and approximately 3 cm³ of muscle tissue was excised and promptly immersed in 10% buffered formalin for subsequent histological evaluation. Subsequently, the biopsy site was sutured, starting with closure of the skeletal muscle using absorbable suture Catgut chromic No. 0 (Atramat, Bragança Paulista, SP, Brazil) and later a non-absorbable suture for the skin. Post-biopsy treatments included the application of penicillin-based antibiotic (Diclope^®^, JA Saúde Animal, Patrocínio Paulista, SP, Brazil) and flunixin meglumine as an analgesic/anti-inflammatory (Niglumine, Hertape-Calier Saude Animal S.A., Juatuba, MG, Brazil) for four consecutive days. Skin sutures were removed two weeks after the biopsy. Muscle tissue was dehydrated through graded ethanol (80, 85, 90, 95, and 100%) and cleared in two changes of xylene. Samples were embedded in paraffin, sectioned at 5 μm on a rotary microtome (RM 2265; Leica Biosystems, Nussloch, Germany), and stained with Masson’s trichrome (Foidart et al. 1981). For the analysis of muscle fiber number and area, 5 digital images of muscle sections were captured from each animal using a 20x magnification. The images were then analyzed using ImageJ software (National Institutes of Health, Bethesda, MD, USA).

### Statistical analysis

The experiment followed a randomized complete block design, using initial SBW as the blocking criterion. Cows were allocated to the two herbage allowance treatments (LHA and HHA) within six blocks (replicates) across 12 paddocks (two paddocks per block). All statistical analyses were performed in SAS 9.4 (SAS Institute, Cary, NC). Residuals from the fitted models were obtained using the OUTPUT statement. Normality was evaluated using the Shapiro–Wilk test (α = 0.05) in PROC UNIVARIATE. Homogeneity of variances was assessed by analyzing squared residuals from the fitted models under the same fixed and random effects structure used for each original response variable. Pregnancy rate was analyzed using a generalized linear mixed model in PROC GLIMMIX, assuming a binomial distribution with a logit link function, with fixed effects evaluated using Wald tests. Quantitative and chemical pasture variables were analyzed using a randomized complete block repeated-measures model with between-paddock variability (Model 1):

$$\begin{aligned}Y_{ijt}&=\mu+HA_j+Per_{t}+(HA \times Per)_{jt}+B_i+P_{ij}+e_{ijt}\end{aligned}$$

where Y_ijt_ is the pasture response in paddock _ij_ at period _t_; µ is the overall mean; HA_j_ is the fixed effect of herbage allowance; Per_t_ is the fixed effect of period; (HA × Per)_jt_ is the fixed interaction; B_i_ is the random effect of block; P_ij_ is the random effect of paddock nested within block × HA (experimental unit; between-paddock variability); and e_ijt_ is the within-paddock residual across periods. For pasture-related responses, HA, period, and their interaction were included as fixed effects; paddock nested within block × treatment was specified as the subject in the REPEATED statement, and the covariance structure was selected based on the lowest Bayesian Information Criterion. Whenever significant effects involving period were detected, the results are presented by period, with values shown for each period in which differences were observed.

Cow performance, carcass traits, and milk yield and composition measured repeatedly over time were analyzed using a randomized complete block repeated-measures model with between- and within-paddock variability (Model 2):

$$\begin{aligned} Y_{ijkt}&=\mu+HA_{j}+Per_t+(HA \times Per)_{jt}+B_{i}+P_{ij}+A_{k(ij)}+e_{ijkt}\end{aligned}$$

where Y_ijkt_ is the response of animal _k_ in paddock _ij_ at period _t_; µ is the overall mean; HA_j_, Per_t_, and (HA × Per)_jt_ are fixed effects; B_i_ and P_ij_ are random effects; A_k(ij)_ is the random effect of animal nested within paddock (within-paddock variability); and e_ijkt_ is the residual error across repeated observations within animal. For animal-level repeated responses, HA, period, and their interaction were included as fixed effects; animal nested within paddock was specified as the subject in the REPEATED statement, consistent with the structure of the dataset, and the covariance structure was selected based on the lowest Bayesian Information Criterion. Whenever significant effects involving period were detected, the results are presented by period, with values shown for each period in which differences were observed.

Calf performance, blood variables of cows and calves, and muscle histology were analyzed as single-time measurements using a randomized complete block model with between- and within-paddock variability (Model 3):

$$Y_{ijk}=\mu+HA_j+B_i+P_{ij}+\beta(x_{ijk}-\overline{X})+\varepsilon_{ijk}$$

where Y_ijk_ is the observation from animal _k_ in paddock _ij_; µ is the overall mean; HA_j_ is the fixed effect of herbage allowance; B_i_ is the random effect of block; P_ij_ is the random effect of paddock nested within block × HA (experimental unit; between-paddock variability); ε_ijk_ is the within-paddock (animal-level) residual; and β(x_ijk_ − x̄) was included only when covariates were used. For cow blood variables at d -11, the corresponding d -151 values were included as covariates. These mixed-model analyses were performed using PROC MIXED (α = 0.05).

When treatment × day interactions were significant, treatment effects were compared within each period using F-tests of simple effects in PROC MIXED (α = 0.05).

## Results

### Herbage measurements

**Fig. 3:**
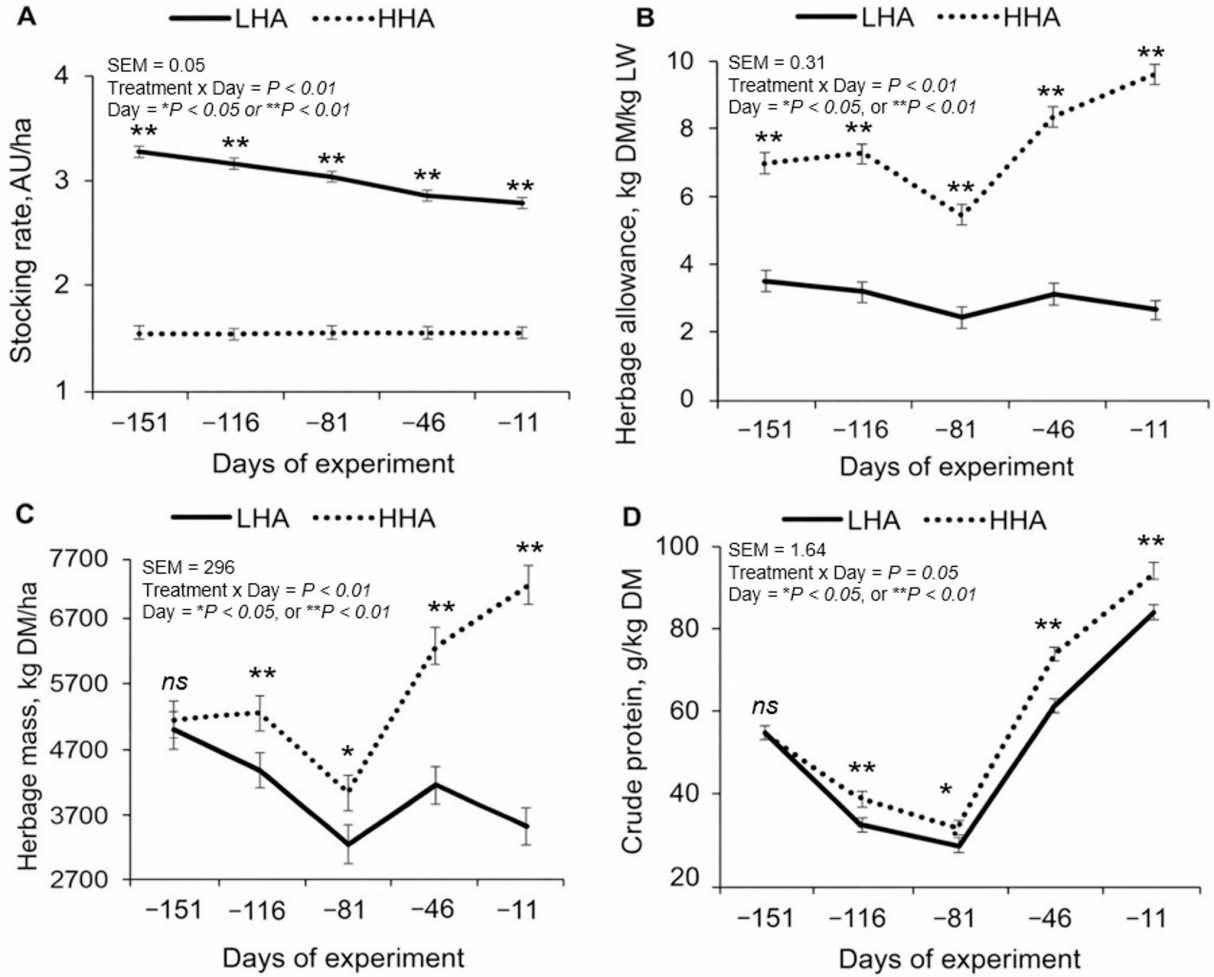
Effects of herbage allowance on *Urochloa brizantha* cv. Marandu on stocking rate (**A**), herbage allowance (**B**), herbage mass (**C**), and herbage crude protein (**D**) over the day

In accordance with the established study protocol, LHA was characterized by higher stocking rate (Fig. 3A; AU/ha) and lower herbage allowance (Fig. 3B; kg DM/kg BW) than HHA across all experimental periods (*P* < 0.01). Except for these variables, the remaining quantitative components and chemical composition did not differ at the beginning of the study (*P* = 0.72) between HHA and LHA (Table 1).

**Table 1:** Quantitative, morphological, and chemical evaluation of pasture grazed by beef cows managed under different herbage allowance levels during mid- to late gestation

Days of experiment^1^ Treatments	-151		-116		-81		-46		-11		SEM	*P*-value^2^		
	LHA	HHA	LHA	HHA	LHA	HHA	LHA	HHA	LHA	HHA		Treatment	Day	Treatment x Day
Herbage characteristics														
Canopy height, cm	40.8	42.2	35.6	35.2	32.2	32.1	26.2	31.6	23.0	31.1	1.50	< 0.01	< 0.01	< 0.01
Total green mass, kg/ha	2166	2187	1012	1416	498	696	910	2398	1161	3145	113	< 0.01	< 0.01	< 0.01
Green leaf mass, kg/ha	942	1033	55.0	181	46.0	57.0	408	1125	674	1674	65.0	< 0.01	< 0.01	< 0.01
Density, kg DM/m³	1.32	1.34	1.20	1.35	1.92	2.29	1.47	1.82	1.66	2.24	0.11	0.03	< 0.01	0.18
Green leaf, %	18.5	20.3	1.17	3.67	1.33	1.50	10.0	17.8	19.0	22.9	0.90	< 0.01	< 0.01	< 0.01
Green stem, %	25.1	22.5	22.3	23.2	13.5	15.7	12.5	20.2	14.5	21.6	1.50	0.03	< 0.01	0.05
Leaf senescence, %	22.8	24.5	18.8	32.5	19.8	38.8	4.00	10.8	4.14	12.9	1.29	< 0.01	< 0.01	< 0.01
Stem senescence, %	33.6	32.7	57.7	40.3	65.5	44.0	73.5	50.8	64.0	45.3	1.58	< 0.01	< 0.01	< 0.01
Green leaf: Green stem Ratio	0.74	0.90	0.05	0.16	0.10	0.10	0.80	0.88	1.31	1.06	0.08	0.13	< 0.01	0.10
Chemical composition														
Dry matter, g/kg	433	402	806	647	671	675	334	319	303	317	15.6	< 0.01	< 0.01	< 0.01
Ash, g/kg DM	57.9	59.0	58.1	63.5	46.6	52.1	64.1	70.8	74.6	68.0	2.44	0.19	< 0.01	0.11
Organic matter, g/kg DM	942	941	942	936	953	948	936	929	924	932	2.43	0.24	< 0.01	0.07
Crude fat, g/kg DM	10.9	11.9	7.30	10.2	3.75	4.74	8.53	10.2	13.1	12.0	1.23	0.18	< 0.01	0.67
NDF, g/kg DM	722	720	804	769	846	826	763	711	720	711	9.70	< 0.01	< 0.01	0.15
Lignin, g/kg DM	59.3	54.6	71.7	63.9	99.6	82.0	90.4	80.9	70.1	67.8	5.23	0.02	< 0.01	0.65
*In vitro DM digestibility*,* g/kg DM*	488	505	347	427	307	366	443	481	500	605	24.7	< 0.01	< 0.01	0.17
iNDF, g/kg DM	201	186	243	226	334	281	253	194	167	152	9.75	< 0.01	< 0.01	0.01
NDIP, g/kg CP	370	351	372	317	353	352	321	272	245	224	15.6	0.01	< 0.01	0.50
ADIP, g/kg CP	71.6	76.1	142	105	182	141	70.6	57.4	49.4	40.4	4.26	< 0.01	< 0.01	< 0.01

Abbreviations: LHA = low herbage allowance; HHA = high herbage allowance; SEM = standard error of the mean; DM = dry matter; NDF = neutral detergent fiber; iNDF = indigestible neutral detergent fiber; NDIP = neutral detergent insoluble protein; ADIP = acid detergent insoluble protein; CP = Crude protein

^1^ Treatments were applied during the last 151 days of gestation, and experimental days are expressed relative to calving

^2^ Statistical significance was declared at *P* < 0.05

There was a treatment × day interaction (*P* ≤ 0.05) for herbage mass (Fig. 3C; kg/ha), leaf senescence (%), total green mass (kg/ha), green leaf (%), green stem (%), canopy height (cm), green leaf mass (kg/ha), and stem senescence (%) (Table 1). Herbage mass and leaf senescence differed between treatments on d -116 (*P* < 0.01), d -81 (*P* < 0.01), d -46 (*P* < 0.01), and d -11 (*P* < 0.01), with higher values observed in HHA compared to LHA. Total green mass and green leaf differed on d -116 (*P* < 0.01), d -46 (*P* < 0.01), and d-11 (*P* < 0.01), with higher values in HHA compared to LHA. Green stem, canopy height, and green leaf mass also differed between treatments on d -46 (*P* < 0.01) and d -11 (*P* < 0.01), with higher values in HHA compared to LHA. In contrast, stem senescence was higher in LHA compared to HHA on d -116 (*P* < 0.01), d -81 (*P* < 0.01), d -46 (*P* < 0.01), and d -11 (*P* < 0.01).

The chemical composition of the herbage showed a treatment × day interaction for CP content (Fig. 3D; g/kg DM), DM content (g/kg herbage mass), iNDF content (g/kg DM), and ADIP content (g/kg CP) (*P* ≤ 0.05; Table 1). CP content differed between treatments on d-116 (*P* < 0.01), d-46 (*P* < 0.01), and d-11 (*P* < 0.01), with higher values in HHA compared to LHA. DM content differed on d-116 (*P* < 0.01), with higher values in LHA compared to HHA. iNDF content differed on d-81 (*P* < 0.01) and d-46 (*P* < 0.01), with higher values in LHA compared to HHA. Likewise, ADIP content differed on d-116 (*P* < 0.01), d-81 (*P* < 0.01), and d-46 (*P* < 0.01), with higher values in LHA compared to HHA.

Among the quantitative herbage components, a main effect of HA (*P* = 0.03) was observed for greater sward density (kg DM/m³) under HHA compared to LHA. Likewise, among the chemical herbage composition, a main effect of HA *(P* < 0.01) was observed for greater in vitro DM digestibility (g/kg DM) under HHA compared with LHA. However, a main effect of HA (*P* ≤ 0.02) was also observed, with lower concentrations of NDF (g/kg DM), lignin (g/kg DM), and NDIP (g/kg CP) for HHA compared with LHA (Table 1).

### Growth performance and carcass ultrasound characteristics in cows

There was a treatment × day interaction (*P* ≤ 0.02) for most cow performance and carcass ultrasound characteristics, except for rump fat thickness (*P* = 0.49; Table 2). No significant differences were observed at d -151 between HHA and LHA for BW (*P* = 0.66), BCS (*P* = 0.30), LMA (*P* = 0.32), and SFT (*P* = 0.45). However, cows maintained in LHA exhibited a 43 kg reduction in BW at d -11 (*P* < 0.001), with no significant difference at d 240 (*P* = 0.12) compared to those maintained in HHA. Accordingly, ADG was lower in LHA cows at d -11 (*P* < 0.001; absolute difference = 0.293 kg/d) but greater at d 240 (*P* < 0.001; absolute difference = 0.136 kg/d) compared with HHA cows. Following the ADG pattern, cows in LHA showed a 0.40-point reduction in BCS at d -11 (*P* < 0.001), which decreased to 0.19 points at d 240 (*P* < 0.001) compared to those in HHA. Cows maintained in LHA exhibited reductions of 9.57% in LMA (*P* = 0.002) and 21.5% in SFT (*P* = 0.02) at d -11, with no differences observed in LMA (*P* = 0.89) and SFT (*P* = 0.92) at d 240 compared to cows in HHA.

**Table 2:** Effects of herbage allowance on performance and carcass characteristics of grazing beef cows during mid- to late gestation

Item	Treatments	Days of experiment^1^			SEM	*P-*value^2^
		-151	-11	240		
Body weight, kg	LHA	443	415	417	3.86	< 0.001
	HHA	445	458	426		
	SEM	5.38	5.37	5.38		
	*P-value* ^*3*^	0.66	< 0.001	0.12		
Average daily gain, kg/d	LHA	-	− 0.200	0.008	0.04	< 0.001
	HHA	-	0.093	− 0.128		
	SEM	-	0.038	0.036		
	*P-value*	-	< 0.001	< 0.001		
Body score condition	LHA	3.63	2.96	3.02	0.06	< 0.001
	HHA	3.69	3.36	3.21		
	SEM	0.06	0.05	0.05		
	*P-value*	0.30	< 0.001	< 0.001		
*Longissimus* muscle area, cm²	LHA	58.1	51.0	53.4	1.06	< 0.001
	HHA	58.5	56.4	51.2		
	SEM	2.27	2.19	2.30		
	*P-value*	0.32	0.002	0.89		
Subcutaneous fat thickness, mm	LHA	3.92	3.11	3.27	0.15	0.02
	HHA	3.87	3.96	3.43		
	SEM	0.41	0.22	0.36		
	*P-value*	0.45	0.02	0.92		
Rump fat thickness, mm	LHA	6.47	4.96	4.83	0.27	0.49
	HHA	6.60	5.57	4.82		
	SEM	0.74	0.61	0.64		
	*P-value*	0.60	0.15	0.85		

Abbreviations: LHA = low herbage allowance; HHA = high herbage allowance; SEM = standard error of the mean

^1^ Treatments were applied during the last 151 days of gestation, and experimental days are expressed relative to calving

^2^Statistical significance for the treatment × day interaction was declared at *P* < 0.05

^3^Statistical significance was declared for each day at *P* < 0.05

### Pregnancy rates, milk yield, and composition

Herbage allowance did not affect overall pregnancy rate (66.7% vs. 65.5% for LHA compared with HHA; *P* = 0.90). Furthermore, there were no effects of treatment (*P* ≥ 0.20) or treatment × day interaction (*P* ≥ 0.70; Table 3) on milk yield or milk composition.

**Table 3:** Effects of herbage allowance during mid- to late gestation on milk yield and composition of Nellore cows during lactation

Days of experiment^1^ Treatments	30		120		210		SEM	*P*-value^2^		
	LHA	HHA	LHA	HHA	LHA	HHA		Treatment	Day	Treatment x Day
Milk yield, kg/d	6.04	5.76	4.52	4.32	3.59	3.43	0.46	0.48	< 0.01	0.97
Fat-corrected milk yield, kg/d	5.63	5.46	4.67	4.55	3.76	3.75	0.48	0.70	< 0.01	0.98
Milk fat, %	3.49	3.53	4.29	4.23	4.49	4.51	0.24	0.98	< 0.01	0.94
Milk protein, %	3.29	3.38	3.78	3.67	3.74	3.64	0.09	0.71	< 0.01	0.20
Milk lactose, %	4.88	4.85	4.52	4.64	4.47	4.40	0.10	0.93	< 0.01	0.44
Milk total solids, %	12.6	12.7	13.7	13.6	13.6	13.6	0.27	0.91	< 0.01	0.86
Milk casein, %	2.58	2.66	3.04	2.95	2.97	2.87	0.08	0.70	< 0.01	0.23
Somatic cells contain, x mil/ml	214	214	351	303	117	147	98.2	0.94	0.07	0.87
Milk urea, mg/dL	9.15	9.04	11.8	11.8	11.7	11.4	0.56	0.79	< 0.01	0.89

Abbreviations: LHA = low herbage allowance; HHA = high herbage allowance; SEM = standard error of the mean

^1^Treatments were applied during the last 151 days of gestation, and experimental days are expressed relative to calving

^2^Statistical significance was declared at *P* < 0.05

### Blood characteristics in cows

cows managed under LHA had greater (*P* < 0.01) urea concentration than those under HHA (Table 4). There was no effect of treatment on the remaining blood characteristics (*P* ≥ 0.12; Table 4).

**Table 4:** Effects of herbage allowance on blood characteristics of grazing beef cows during mid- to late gestation

Item	Treatments^1^		SEM	*P*-value^2^
	LHA	HHA		
Insulin, ulU/mL	13.1	14.4	1.97	0.63
IGF-1, ng/mL	259	264	27.5	0.89
Glucose, mg/dL	83.9	89.7	6.11	0.43
Urea, mg/dL	30.0	25.8	1.83	< 0.01
Albumin, g/dL	3.62	3.90	0.12	0.09
Creatinine, mg/dL	1.99	2.09	0.07	0.26
Total protein, g/dL	8.31	8.98	0.26	0.07
Cholesterol, mg/dL	139	152	18.4	0.31
Triglycerides, mg/dL	19.2	18.8	1.12	0.79
Amino aspartate-transferase, U/L	72.2	71.9	2.65	0.93
Gamma-glutamyl transferase, U/L	15.5	15.4	0.81	0.93

Abbreviations: LHA = low herbage allowance; HHA = high herbage allowance; SEM = standard error of the mean; IGF-1 = Insulin-like Growth Factor 1

^1^ Treatments were applied during the last 151 days of gestation, experimental days are expressed relative to calving, and the results correspond to the last 11 days of gestation (d-11)

^2^ Statistical significance for the treatment effect was declared at *P* < 0.05

### Growth performance in calves

An effect of HA was observed (*P* < 0.01), with calves from cows maintained under HHA conditions showing greater body weight at birth, d 120, and at weaning compared with calves from LHA cows. A main effect of HA was also detected for ADG at d 120 (*P* = 0.03), with greater ADG in calves from HHA cows than in those from LHA cows. However, no effect of HA was observed for ADG at weaning (*P* = 0.64; Table 5).

**Table 5:** Effects of herbage allowance during mid- to late gestation on offspring performance in grazing beef cows

Item	Treatments^1^		SEM	*P*-value^2^
	LHA	HHA		
Body weight, kg				
Birth weight	34.9	39.1	1.35	< 0.01
d 120	123	136	3.65	< 0.01
Weaning^3^	174	188	5.24	< 0.01
Average daily gain, kg/d				
d 120	0.739	0.800	0.02	0.03
Weaning	0.601	0.614	0.03	0.64

Abbreviations: LHA = low herbage allowance; HHA = high herbage allowance; SEM = standard error of the mean

^1^ Treatments were applied during the last 151 days of gestation, and experimental days are expressed relative to calving

^2^ Statistical significance for the treatment effect was declared at *P* < 0.05

^3^ Weaning weight adjusted to 205 days

### Blood characteristics in calves

The blood AST concentration of calves from the LHA was higher (*P* = 0.05), with a 14.1% increase on d 120, but no difference was observed (*P* = 0.14) on weaning compared to calves from the HHA. No differences were detected on d 120 and d 240 for characteristics such as insulin, IGF-1, glucose, urea, albumin, creatinine, total protein, cholesterol, triglycerides, and GGT (*P* ≥ 0.11; Table 6).

**Table 6:** Effects of herbage allowance during mid- to late gestation on offspring blood characteristics in grazing beef cows

Item	Days of experiment^1^	120		SEM	240		SEM	*P*-value^2^	
	Treatments	LHA	HHA		LHA	HHA		120	240
Insulin, ulU/mL		10.9	9.50	2.98	8.51	8.77	1.61	0.72	0.90
IGF-1, ng/mL		227	226	27.5	133	147	7.79	0.99	0.21
Glucose, mg/dL		122	114	4.97	91.1	86.0	2.57	0.18	0.12
Urea, mg/dL		12.8	13.8	1.61	5.24	4.35	0.60	0.64	0.20
Albumin, g/dL		4.23	4.34	0.14	3.91	3.64	0.21	0.46	0.23
Creatinine, mg/dL		1.87	1.98	0.11	1.98	1.89	0.05	0.30	0.22
Total protein, g/dL		8.06	8.28	0.26	7.67	7.44	0.14	0.44	0.11
Cholesterol, mg/dL		180	180	18.4	195	180	9.54	0.97	0.13
Triglycerides, mg/dL		24.9	23.5	2.96	25.3	23.4	2.03	0.66	0.33
Amino aspartate-transferase, U/L		92.3	79.3	5.49	69.0	65.0	2.47	0.05	0.14
Gamma-glutamyl transferase, U/L		21.0	18.5	1.86	13.7	12.9	0.98	0.26	0.52

Abbreviations: LHA = low herbage allowance; HHA = high herbage allowance; SEM = standard error of the mean; IGF-1 = Insulin-like Growth Factor 1

^1^ Treatments were applied during the last 151 days of gestation, and experimental days are expressed relative to calving

^2^ Statistical significance for the treatment effect was declared at *P* < 0.05

### Number and area of muscle fibers in calves

**Fig. 4:**
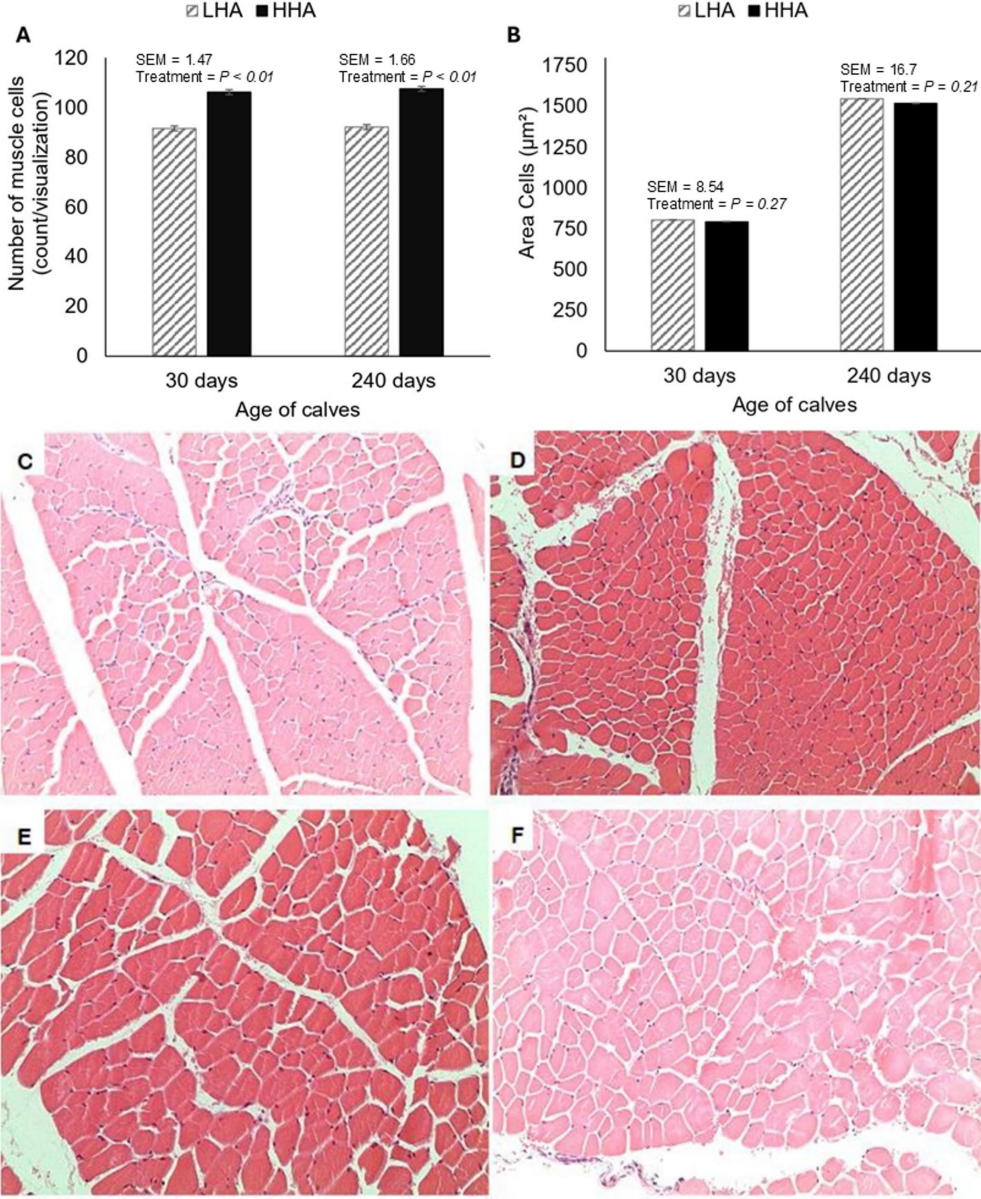
Effects of herbage allowance during mid- to late gestation in grazing pregnant beef cows on offspring skeletal muscle fiber number (**A**) and area (**B**). Representative histological images of skeletal muscle in calves from low herbage allowance at 30 days of age (**C**), high herbage allowance at 30 days of age (**D**), low herbage allowance at 240 days of age (**E**), and high herbage allowance at 240 days of age (**F**)

There were significant treatment effects on the number of muscle fibers at 30 days after birth (*P* < 0.01; 91.6 vs. 106 counts/visualization) and at 240 days after birth (*P* < 0.01; 92.1 vs. 107 counts/visualization; Fig. 4A). However, no differences were observed between calves from LHA and HHA cows regarding muscle fiber area at 30 days after birth (*P* = 0.27; mean: 799 μm²) or at 240 days after birth (*P* = 0.21; mean: 1533 μm²; Fig. 4B).

## Discussion

This study successfully established two distinct nutritional conditions for pregnant Nellore cows from mid- to late gestation by manipulating stocking rate, which effectively resulted in contrasting herbage allowances under continuous grazing conditions. Low herbage allowance promotes a typical grazing behavioral pattern in cattle, characterized by reduced bite size, impaired herbage apprehension, and, consequently, increased locomotion during grazing, which reduces the effective time allocated to intake (Chacon and Stobbs 1976; Hodgson 1990; Yu et al. 2024; Slayi and Jaja 2025). However, increased grazing time and locomotion do not fully compensate for this limitation, resulting in reduced dry matter intake and, consequently, impaired performance (Trindade et al. 2012; Hou et al. 2023). In addition to this nutritional restriction, potential negative direct and carry-over effects may occur in the progeny when pregnant cows are subjected to such conditions (Funston et al. 2010; Claramunt et al. 2024). These factors help to explain the observed outcomes in cows maintained under LHA from mid- to late gestation, which exhibited lower BW, ADG, BCS, LMA, and SFT as a consequence of reduced herbage allowance. Nevertheless, proper pasture management, with balanced herbage supply, results in improved animal performance, as it has been estimated that between 60% and 90% of the variation in grazing animal performance is explained by herbage availability, which allows selective intake of more nutritious plant portions (Sollenberger et al. 2005; Sollenberger and Vanzant 2011). Even under the low herbage quantity and quality conditions observed in the present study, cows under HHA maintained better performance, with no reductions observed in BW, ADG, LMA, or SFT, likely because the greater herbage allowance enabled selective intake of more nutritious pasture components (Trindade et al. 2016; Do Carmo et al. 2021; Sousa et al. 2024).

In contrast, BW, ADG, LMA, and SFT were not negatively affected in HHA cows during gestation, likely due to selective feeding mechanisms in areas with greater herbage mass. Understanding how pregnant cows cope with nutritional restrictions requires consideration of the interaction between homeostasis and homeorhesis. While homeostasis maintains internal physiological balance, homeorhesis prioritizes functions such as maintenance or gestation-lactation (Bauman and Currie 1980). The reduction in LMA and SFT in LHA cows indicated mobilization of body tissues, a distinct condition from that observed in HHA cows. This suggests that insufficient nutrient supply resulted in greater mobilization of body reserves in LHA cows (Lopes et al. 2020). Additionally, under restricted conditions, amino acid oxidation is intensified to supply energy to the placenta during gestation (Vaughan et al. 2017; Meneses et al. 2024). Consequently, increased mobilization of amino acids from muscle tissue to hepatic gluconeogenesis results in elevated blood urea concentrations due to amino acid deamination (Wood et al. 2013; Lopes et al. 2020). These findings align with the present study, where LHA cows exhibited 14.0% higher blood urea concentrations than HHA cows, highlighting muscle tissue degradation to maintain homeorhesis. The mobilization of SFT observed in nutritionally restricted cows, such as those in LHA, may be associated with the utilization of lipids as an energy source due to altered skeletal muscle metabolism, favoring fatty acid oxidation over carbohydrate utilization (Schäff et al. 2013; Jennings et al. 2016).

During lactation, HHA cows experienced reductions in BW, ADG, BCS, LMA, and SFT between d -11 and d 240; however, by d 240, these values were statistically similar to those of cows maintained under LHA. One possible explanation is that HHA cows exhibited higher BW and BCS at calving compared to LHA cows. According to Ayres et al. (2014), cows in better body condition at calving experience greater postpartum body reserve losses due to higher energy reserves and BW, leading to increased maintenance energy demands (Chizzotti 2008; NASEM 2016). By contrast, milk yield was not altered by differences in herbage availability during gestation. In dairy cattle, greater prepartum nutrient intake has been linked to variable effects on milk yield and composition (Chew et al. 1984; Bell et al. 2000; Kokkonen 2014), whereas such responses are seldom evident in beef cows. For example, Larson et al. (2009) detected no change in milk yield when beef cows received 0.45 kg/d of a 28% CP supplement during late gestation. Likewise, Valiente et al. (2018) reported that Angus cow milk yield and composition were unaffected by dietary CP (6% vs. 12%) from mid-gestation to calving, and noted that postpartum nutrition can offset adverse gestational nutrition effects on milk yield. In dairy cows, postpartum loss of body condition reflects negative energy balance due to the high demands of maintenance and milk production exceeding intake (Parr et al. 2015; Štolcová et al. 2024). In beef cows, however, this negative energy balance is less pronounced due to their lower milk yield potential (Hart et al. 1975). Similar condition may have occurred in the present study, as all cows were managed on the same pasture after calving, and the first milk sampling was performed 30 days postpartum, which is likely a sufficient period to attenuate the nutritional effects of gestation on milk yield and composition. Furthermore, the nutrient level in the postpartum diet can compensate for the effects of gestational restriction on milk yield and composition (Bell et al. 2000). Consequently, despite a 0.40 points in BCS difference in d -11 between LHA and HHA cows, no effects on milk yield or composition were observed. These findings are supported by data indicating similar milk yield in beef cows with different body condition scores during gestation, since management after calving has a greater influence on milk production in beef cows (Spitzer et al. 1995; Marques et al. 2016).

All previously observed and mentioned conditions are consistent with findings in the progeny of cows maintained under LHA and HHA conditions. At birth, at d 120, and at weaning, calves from HHA cows exhibited higher BW and greater ADG at d 120 compared to calves from LHA cows. These effects were likely primarily associated with maternal nutrition during gestation, since calves from HHA cows already exhibited greater BW at birth and no differences were observed in milk yield or composition during the lactation phase. Nevertheless, a potential contribution of calf herbage intake cannot be ruled out. These results align with those of Marques et al. (2016), who found no differences in milk yield among Angus x Hereford crossbred cows with varying BCS during gestation. However, they observed that calves from cows that gained BCS in mid and late gestation had higher weaning weights, an effect attributed to intrauterine development driven by maternal nutrition. Additionally, greater skeletal muscle cell hyperplasia was observed in calves from HHA cows, approximately 13.7% higher than in calves from LHA cows. This factor explains the higher BW from birth to weaning, as well as the increased ADG at d 120 in calves from HHA cows. In this context, Du et al. (2010) explain that cellular-level modifications during gestation influence muscle fiber hyperplasia, occurring until the seventh month of gestation, and muscle fiber hypertrophy, which begins in the last trimester of gestation and extends throughout life. However, no differences in muscle fiber hypertrophy were observed between calves from LHA and HHA cows. Similar results were reported by Costa et al. (2021b), in which Tabapuã calves from protein-supplemented cows (40% CP) during mid-gestation showed a greater number of muscle fibers at 30 and 450 days of age, with no differences in cell area, compared with calves from non-supplemented cows. Similarly, Márquez et al. (2017) observed that protein supplementation (30% CP) of Nellore cows during mid and late gestation resulted in a higher number of muscle fibers at 30 days of age in calves, without differences in muscle fiber cell area compared to calves from cows subjected to dietary restriction.

However, the ADG of calves at weaning may have been influenced by environmental conditions, as described by Barcellos et al. (2021), who reported that the effects of maternal stimuli during gestation can be long-lasting but require favorable postnatal environments. For example, balanced maternal nutrition can result in offspring that express prolonged productive responses, although the expression of these effects depends on adequate postnatal conditions. It is plausible that the similarity in ADG between treatments at weaning was due to reduced rainfall (Fig. 1), which likely decreased herbage availability and quality after d 120. At this stage, daily milk production of beef cows is already declining, which makes calves more dependent on herbage-based nutrition. Based on these observations, it is important to highlight that Cidrini et al. (2025) published a follow-up study evaluating the effects of maternal nutritional history after weaning, based on the gestational period from d -151 to d 0. In that study, animals originating from HHA cows and maintained under an intensive nutritional regime during the growing and finishing phases produced heavier carcasses and greater LMA compared with animals originating from LHA cows.

Calves from LHA cows exhibited a 14.1% increase in AST levels at d 120 compared to calves from HHA cows, possibly due to hepatic damage resulting from maternal dietary restriction during gestation. Liver function is established during fetal life and receives high priority in maternal nutrient partitioning, consequently, nutritional constraints can impair hepatic growth and function, leading to disturbances in metabolic balance, nutrient handling, and detoxification capacity (Hyatt et al. 2008; Gao et al. 2014). Moreover, during gestation, visceral organs such as the liver are preferentially supplied with nutrients over skeletal muscle (Du et al. 2010). Biochemical tests, such as blood AST measurement, can be employed to assess potential hepatic dysfunction (Ogunkeye and Roluga 2006). In this study, calves from LHA cows exhibited lower BW from birth to weaning compared to HHA calves. The results were reported by Gao et al. (2014) reported that offspring of nutritionally restricted ewes exhibited greater serum AST concentrations compared with offspring of ewes without nutritional restriction during late gestation. However, isolated AST assessment is insufficient to determine hepatic dysfunction or damage in progeny from cows under LHA conditions. This finding highlights the need for further investigation into the effects of nutritional restriction on calf liver function, contributing to the understanding of their lower performance.

## Conclusion

Adequate herbage allowance during mid- to late gestation enhances maternal performance and metabolic status, supporting efficient tissue reserve dynamics that contribute to fetal skeletal muscle development. These prenatal effects carry over into postnatal life, enhancing offspring growth performance through weaning. Our findings indicate that nutritional management based on herbage allowance is a key strategy for optimizing cow–calf productivity in beef production systems.

## Data Availability

The dataset supporting the conclusions of this article is not stored in an official repository but can be obtained upon request from the corresponding authors.
